# Mechanistic insights into Circ-MBOAT2-mediated regulation of TLK1 through miR-664b-3p in non-small cell lung cancer

**DOI:** 10.1186/s41065-025-00439-y

**Published:** 2025-05-14

**Authors:** DanTing Zhao, Cong Wang, GuangCheng Zhang, ZhengChang Song, ChunYu Luan

**Affiliations:** Department of Respiratory and Critical Care Medicine, The Second Affiliated Hospital of Shandong First Medical University, No. 366 Taishan Street, Taishan District, Tai’an City, Shandong Province 271000 China

**Keywords:** Non-small cell lung cancer, Circ-MBOAT2, TLK1, miR-664b-3p, EMT, Proliferation

## Abstract

**Background:**

Emerging evidence highlights the critical involvement of dysregulated circular RNAs (circRNAs) in non-small cell lung cancer (NSCLC) pathogenesis. Nevertheless, the precise functional role and mechanistic contributions of circ-MBOAT2 in NSCLC remain poorly characterized. The purpose of this study was to investigate the pathogenesis of NSCLC based on circ-MBOAT2.

**Methods:**

Our investigation focused on the interplay among circ-MBOAT2, miR-664b-3p, and Tousled-like kinase 1 (TLK1) mRNA in NSCLC tissues, along with their association with the clinical and pathological characteristics of NSCLC patients. Sequences or plasmids were transfected into A549 cells. Gene expressions were identified using RT-qPCR and Western blot analysis. NSCLC cells’ cancerous characteristics were identified using CCK-8, EdU, AnnexinV-PI double staining, and Transwell, while their in vivo growth was assessed through a xenografted tumor assay. To monitor alterations in the CD8^+^ T cell ratio and inflammatory factors in PBMCs, co-cultures were created with both normal human PBMCs and A549 cells. Evaluations using bioinformatics software, dual luciferase reporter tests, and RIP assays were performed to verify the connection between circ-MBOAT2 and miR-664b-3p, as well as the interaction between miR-664b-3p and TLK1.

**Results:**

Circ-MBOAT2 expression was up-regulated in NSCLC, and reducing circ-MBOAT2 hampered NSCLC cell proliferation, EMT, immune escape, and tumor growth in vivo. There was a negative correlation between miR-664b-3p expression and circ-MBOAT2, and miR-664b-3p could compete with circ-MBOAT2 for binding. miR-664b-3p downregulation impaired the anti-tumor effect of circ-MBOAT2 reduction on NSCLC cells. TLK1 expression was elevated in NSCLC specimens compared to adjacent normal tissues (*p* < 0.001), negatively correlated with miR-664b-3p (*r*=-0.351, *p* < 0.001), and positively correlated with circ-MBOAT2 (*r* = 0.341, *p* < 0.001). In vitro functional experiments showed that silencing TLK1 restrained NSCLC cell proliferation, EMT, and immune escape, whlie TLK1 overexpression rescued the inhibitory effects of miR-664b-3p on NSCLC cell malignant behaviors.

**Conclusion:**

Circ-MBOAT2 promotes NSCLC cell proliferation, EMT and immune escape by competitively binding to miR-664b-3p to promote TLK1 expression.

## Introduction

Lung cancer continues to be the top cause of cancer-related deaths worldwide, with non-small cell lung cancer (NSCLC) making up almost 85% of all lung cancer cases [1]. Recent epidemiological projections indicate that in 2024, the United States will witness approximately 234,580 new lung cancer diagnoses and 125,070 associated deaths, accounting for 20.4% of all cancer mortality. Despite substantial progress in therapeutic development, early detection technologies, and staging system refinements, the five-year survival rate for pulmonary malignancy patients remains persistently below 25%, highlighting the imperative need for novel prognostic biomarkers and precision treatment strategies [2].

Current therapeutic approaches for early-stage NSCLC consider surgical resection and chemotherapy as primary treatment modalities. For surgically ineligible patients, multimodal regimens integrating concurrent chemoradiation with immune checkpoint inhibitors have shown significant survival advantage compared to monotherapy approaches [3, 4]. The progressive elucidation of cancer’s biological complexity has catalyzed therapeutic innovation, while immune surveillance evasion persists as a fundamental oncogenic hallmark that continues to challenge treatment efficacy [5]. Tumor immune escape occurs when tumor cells evade immune recognition and attack, enabling continued invasion and proliferation [6]. Immune escape is a necessary step in the transformation of lung tumor precursor cells to tumor cells, and one that is crucial to determining lung tumor subgroups [7]. The durability of anti-tumor immune response to radiation therapy is hampered by frequent immune escape, which usually occurs with tumor recurrence [8].

Non-coding RNAs known as circRNAs are generated through the reverse splicing of pre-mRNAs, leading to a covalently closed loop structure [9]. This unique closed-loop structure confers enhanced ribonuclease resistance and superior structural stability compared to linear transcripts, thus making it a potential clinically valuable biomarker for non-invasive diagnosis, monitoring of therapeutic response, and prognosis assessment in cancer [10, 11]. Additionally, emerging evidence highlights the pivotal role of circRNAs in mediating therapeutic resistance in NSCLC [12]. For instance, circ_PPAPDC1, a circRNA that is significantly upregulated in Osimertinib-resistant NSCLC specimens, has been reported to enhance the malignant phenotype of resistant cancer cells by sponging miR-30a-3p [13]. Another study reveals that circKIAA0182 modulates cisplatin resistance through its interaction with the RNA-binding protein YBX1, thus uncovering an alternative circRNA-mediated mechanism of chemoresistance in NSCLC [14]. The circRNA circ-MBOAT2 has been identified as a pro-oncogenic factor in colorectal cancer [15], pancreatic cancer [16], and intrahepatic cholangiocarcinoma [17]. Yet, it is unclear how circ-MBOAT2 effects NSCLC on a molecular level.

CircRNAs have been demonstrated to function as microRNA (miRNA) sponges, thereby modulating the biological activities of miRNAs and contributing to carcinogenesis and tumor progression [18]. As an illustration, circ_0017552 promotes colon cancer progression by influencing the miR-338-3p/NET1 axis [19]. In addition, circ_0004015 inhibits tumor progression and enhances cisplatin chemoresistance in NSCLC by targeting miR-198. In another NSCLC study [20], circ_0087378 demonstrates significant upregulation, exacerbating malignant cell behavior through DDR1 activation via sponging miR-199a-5p [21]. MiRNAs, small non-coding RNAs approximately 22 nucleotides in length, are evolutionarily conserved and play critical roles in post-transcriptional gene regulation. Over the past two decades, extensive research has established the connection between miRNAs and various cancers [22, 23]. Studies on glioma demonstrate that hsa-miR-516a-5p/516b-5p suppresses T98G cell invasion [24]. miR-664b-3p has been implicated in colorectal cancer [25, 26]. Intriguingly, prior investigations indicate that downregulated miR-664b-3p contributes to cisplatin resistance in NSCLC [27]. However, the precise role of miR-664b-3p in NSCLC and its mechanistic interaction with circRNAs remain to be fully elucidated.

Building on bioinformatic evidence suggesting miR-664b-3p as a potential target of circ-MBOAT2, the goal of this study is to elucidate the clinical significance of circ-MBOAT2 in NSCLC and to unravel its molecular mechanisms mediated by miR-664b-3p regulation. Our experimental data demonstrate that circ-MBOAT2 promotes NSCLC cell proliferation, EMT, and immune escape through regulation of the miR-664b-3p/Tousled-like kinase 1 (TLK1) axis. These findings suggest that circ-MBOAT2 may serve as a novel diagnostic biomarker and therapeutic target for NSCLC.

## Materials and methods

### Patients and specimens

Ninety-seven endoscopic biopsy specimens confirmed as NSCLC in The Second Affiliated Hospital of Shandong First Medical University between June 2017 and October 2021 were collected. All included patients had a pathologic diagnosis of NSCLC and had not received any form of radiotherapy or chemotherapy. Exclusion criteria: age > 80 years; combined immune system diseases. Surgically resected NSCLC tissues should be complete, including cancerous tissues and corresponding paracancerous tissues. The paracancerous tissues should be at least 5 cm away from the edge of the tumor, and there should be no cancerous cells left. The resected tissues were frozen in liquid nitrogen and stored in -80℃. All included patients had in-depth understanding of the relevant contents of this study and signed an informed consent form about this study.

All protocols were approved by the Ethics Committee of The Second Affiliated Hospital of Shandong First Medical University, and the clinicopathologic characteristics of all included patients are shown in Table 1.

**Table 1:** Association of circ-MBOAT2 with pathological parameters of NSCLC

Clinical indexes	*n* = 97	Circ-MBOAT2		*P*
		Low (*n* = 49)	High (*n* = 48)	
Gender				0.618
Male	61	32	29	
Female	36	17	19	
Age				0.619
60 or less	57	30	27	
More than 60	40	19	21	
Histological grade				**0.002**
Well and moderate	33	24	9	
Poor	64	25	39	
Tumor size				**0.006**
5 or less	44	29	15	
More than 5	53	20	33	
Tumor stage				**0.017**
T1 + T2	40	26	14	
T3 + T4	57	23	34	
Histological subtype				0.748
Squamous cell carcinoma	55	27	28	
Adenocarcinoma	42	22	20	
Lymphatic metastasis				**0.003**
No	51	33	18	
Yes	46	16	30	

Note: The data in the table were analyzed using the chi-square test, with *P* < 0.05 indicating a statistically significant difference

### Cell culture

Human normal lung epithelial cell line (BEAS-2B) with human NSCLC cell lines NCI-H460, HCC827, NCI-H2228, and A549 were obtained from American Type Culture Collection (Chicago, IL, USA). A549 was cultured in DMEM (Thermo Fisher Scientific, Waltham, MA, USA) and the other cell lines were cultured in RPMI 1640 medium (Thermo Fisher Scientific), both of which contained 10% FBS with penicillin/streptomycin 100 U/mL. There was an every other day renewal of the culture solution. Logarithmic-growing cells were taken for subsequent experiments.

### Cell transfection

A549 cells were transfected with sh-circ-MBOAT2#1, sh-circ-MBOAT2#2, sh-TLK1#1, sh-TLK1#2, sh-NC, miR-664b-3p mimic, mimic-NC, miR-664b-3p inhibitor, inhibitor-NC, pcDNA-TLK1, and pcDNA-NC. The procedure was complied to Lipofectamine 2000 kit (Invitrogen, Waltham, MA, USA). All sequences and plasmids were synthesized and prepared by Genepharma (Shanghai, China).

### RNase R treatment assay

Total RNA was isolated from A549 cells and segregated into two categories: one untreated and the other treated with RNase R (3 U/µg RNA, Geneseed, Guangzhou, China) for half an hour at 37 °C. Subsequently, the RNAs underwent purification via the RNeasy MinElute Cleaning Kit (QIAGEN, Valencia, CA, USA). Changes of circ-MBOAT2 and linear MBOAT2 were detected by RT-qPCR.

### Actinomycin D assay

A549 cells were put in six-well plates at 5 × 10^5^ cells/well, and actinomycin D (Sigma-Aldrich, St. Louis, MO, USA) was added to each well at 2 µg/ml at intervals of 0 h (control), 4 h, 8 h, 12 h, and 24 h. At each time point, RNA expressions were analyzed using RT-qPCR.

### Nucleic acid electrophoresis

A 2% agarose gel electrophoresis was conducted using 45 mmol/L triborate and 1 mmol/L EDTA (TBE). DNA underwent separation through electrophoresis at 120 volts for half an hour, employing marker L (50–500 base pairs) (Beyotime, Shanghai, China). Target bands were located by UV light irradiation.

### RNA FISH-immunofluorescence microscopy

Fluorescent In Situ Hybridzation Kit (RiboBio, Guangzhou, China) was used. The cells underwent fixation using 4% paraformaldehyde for a duration of 10 min at ambient temperature, followed by a 5-minute immersion in PBS with 0.5% Triton X-100 (1 ml) at 4 °C.

Probe assay: Each well received 200 µL of prehybridization solution at 37 °C for half an hour, followed by preheating the solution, and then 2.5 µL (20 µmol/L) of Cy3-circ-MBOAT2 probe (RiboBio) was mixed into 100 µL of the hybridization mixture. The cells underwent hybridization throughout the night in a 100 µL solution of probe hybridization, which included the probe, maintained at 37 °C and shielded from light. Under light-avoidance conditions, the cells underwent a 15-minute DAPI restaining, followed by a 5-minute PBS wash (thrice). The slices were sealed and photographed under fluorescence microscopy.

### Subcellular separation experiments

Nuclei and cytoplasmic components of A549 cells were isolated using PARIS kits (Thermo Fisher Scientific). Cells (2 × 10^6^) were combined with 450 µL of cell isolation buffer. Subsequently compounds were incubated on ice for 5 min and centrifuged at 500 g, 4 °C for 5 min. Supernatant and pellets were used to collect cytoplasmic and nuclear RNAs. The nucleus and cytoplasm, respectively, were referred to by U6 and GAPDH.

### CCK-8

A549 cells underwent two washes using PBS. Following digestion with trypsin (0.25%), the cells were reconstituted to a concentration of 20 cells/µl, with approximately 2 × 10^3^ cells/well being introduced into a 96-well plate. Following cell culture periods of 24, 48, and 72 h, each well received 10 µl of CCK-8 (Dojindo, Tokyo, Japan), with the incubation extending for 3 hours. The absorbance levels in each well were measured using a microplate reader (450 nm).

### EdU

Cells were put into 96-well plates at 6000 cells/well, and continued to incubate for 24 h. When the cell fusion reached 60%, 100 µL of diluted EdU solution (RiboBio) was supplemented to each well, and incubation was continued for 2 h. Fluorescence microscope images were taken in four random fields of view after cells were fixed and stained.

### Flow cytometry

Post 0.25% trypsin digestion (EDTA-free) (Boster, Wuhan, China), A549 cells underwent two rounds of centrifugation. Utilizing the Annexin-V-FITC apoptosis detection kit (Biovision, USA), the Annexin-V-FITC/PI staining mixture was created. A total of 106 cells were reconstituted in 100 µL of the stain solution, left to incubate for 15 min, and then combined with 1 mL of HEPES. FITC and PI fluorescence was identified through 488 nm excitation at 515 nm and 620 nm band-pass filters, in that order, while apoptosis was observed using flow cytometry (BD Biosciences, Franklin Lakes, NJ, USA).

### Transwell

A Transwell-chamber culture system (Becton, Dickinson and Company, USA) was used to test the migratory ability of cells, and a Matrigel-coated Transwell chamber (Becton, Dickinson and Company) was used to test the invasive ability. Then, cells underwent digestion, were cleansed twice using a serum-free culture medium, then resuspended and tallied. To the upper chamber, cells (200 µL, 1 × 10^5^) were introduced, followed by the addition of 500 µL of DMEM with 10% FBS (20 ng/ml) to the lower chamber, where it was incubated at 37 °C for a day. The residual cells on the lower chamber underwent fixation using 4% paraformaldehyde for 10 min, followed by staining with crystal violet and examination through a light microscope. Cell counting was performed using Image J software.

### PBMCs Preparation and co-culture

PBMCs were extracted from the peripheral blood of 30 mL in healthy individuals using the Ficoll lymphocyte separation medium (Hao Yang Biological Manufacture, Tianjin, China). PBMCs at 1 × 10^6^ cells/mL, were grown in RPMI 1640 medium (comprising 10% FBS, 100 µg/mL streptomycin, 100 U/mL penicillin), with the third and fourth PBMC passages being utilized.

PBMCs were activated by 20 µg/mL phytohemhemagglutinin (Sigma-Aldrich) for 72 h. PBMCs were co-cultured with A549 cells at a 5:1 ratio in 96-well plates for 48 h. IFN-γ, TNF-α, and IL-10 in PBMC supernatants were measured by ELISA kits (Abcam, Cambridge, MA, USA), and the proportion of CD8^+^ T cells in PBMCs was detected by flow cytometry (BD Biosciences).

### RT-qPCR

Total RNA was extracted from tissues and cells with TRIzol reagent (Invitrogen), with its concentration and purity measured using a UV spectrophotometer (Thermo Fisher Scientific). The mRNA underwent reverse transcription through the GoldScript one-step RT-PCR Kit (Applied Biosystems, USA). The creation of miRNA cDNA utilized the Hairpin-itTM miRNA Quantification Kit (GenePharma). The PCR process utilized RT-qPCR assay kits (Promega, USA), with the primers crafted by Sangon (Shanghai, China) (Table 2). The procedure for the reaction involved: initial denaturation at 95 °C lasting 5 min, followed by 40 cycles of denaturation at 94 °C for 30 s, annealing at 60 °C for 30 s, and extension at 72 °C for 2 min, culminating in a total extension at 72 °C for 5 min. The comparative expression rates were determined using the 2^−△△Ct^ technique.

**Table 2:** Fluorescent quantitative PCR primer sequences

Genes	Primer Sequence (5′ − 3′)
miR-664b-3p	Forward: 5’-TGGGCTAAGGGAGATGATTG-3’
	Reverse: 5’-TGTAGGCTGGGGAGGCAAA-3’
U6	Forward: 5’-CTCGCTTCGGCAGCACA-3’
	Reverse: 5’-AACGCTTCACGAATTTGCGT-3’
circ-MBOAT2	Forward: 5’-ACCTCACAGTGTGCCAAGTT-3’
	Reverse: 5’-CAAATGGCTGCTAGCAAGGC-3’
linear MBOAT2	Forward: 5’-TGCCCATCGACCAGGTATG-3’
	Reverse: 5’-TTGGCACACTTGAGGTATCC-3’
TLK1	Forward: 5’-ACTGGAAGTACGGGCAGTTG-3’
	Reverse: 5’-CTGTGGGGAGGTTTGCGTTTG-3’
GAPDH	Forward: 5’-GAGTCCACTGGCGTCTTCA-3’
	Reverse: 5’-GGTCATGAGTCCTTCCACGA-3’

Note: MBOAT2, membrane bound O-acyltransferase domain containing 2; TLK1, tousled-like kinase 1; GAPDH, glyceraldehyde-3-phosphate dehydrogenase

### Western blot

Proteins were isolated by RIPA lysis buffer (Zeye, Shanghai, China), and protein concentration was measured by BCA method. The extracted sample protein solution and the lysis buffer were mixed at 2:1, boiled to denaturation, and subjected to SDS-PAGE. The proteins were transferred to PVDF membranes, incubated with primary antibodies against TLK1 (1:1000, 720397, Thermo Fisher Scientific), Ki-67 (1:5000, ab92742, Abcam), Bax (1:1000, ab32503, Abcam), Bcl-2 (1:1000, ab196495, Abcam), E-cadherin (1:500, #3195, Cell Signaling Technology [CST], USA), N-cadherin (1:500, #13116, CST), Vimentin (1:500, #5741, CST), and GAPDH (1:2500, ab9485, Abcam) overnight and then with HRP-labeled secondary antibody (1:5000, Abcam) for 1 h. Proteins were finally developed by ECL chemistry and subjected to gray scale analysis using Image J software.

### Dual luciferase gene reporter assay

The Starbase bioinformatics program identified the binding locations of miR-664b-3p and circ-MBOAT2 or TLK1. Synthesis of circ-MBOAT2 or TLK1 3’UTR promoters, which include a miR-664b-3p binding site, was completed, followed by the creation of either circ-MBOAT2 wild-type (WT) plasmid or TLK13’UTR WT plasmid. Mutations were made to the binding site to create either a circ-MBOAT2 mutant (MUT) plasmid or a TLK1 3’UTR MUT plasmid. The cells underwent inoculation in 96-well plates, followed by the co-transfection of miR-664b-3p mimic or mimic-NC into A549 cells. This was achieved by blending them with circ-MBOAT2/TLK1-WT and circ-MBOAT2/TLK1-MUT plasmids, respectively. Forty-eight hours post-transfection, cells underwent lysis, and the activity of luciferase was identified through a luciferase assay kit (Thermo Fisher Scientific).

### RIP assay

RIP assays were performed with the RIP assay kit (Millipore, Billerica, MA, USA). Cells were lysed using complete RIP lysis buffer, and cell lysates were incubated overnight at 4 °C with spherical beads coated with Ago2 and IgG antibodies overnight. RNA was then extracted using the RNeasy MinElute Cleanup Kit (Qiagen), followed by RT-qPCR analysis.

### In vivo assay

Ten BALB/C nude mice, with 16–20 g, 7–8 weeks old, were purchased from Shanghai SLAC Laboratory Animals Ltd. (Shanghai, China) and fed in an SPF-grade room. Two groups of five mice each were formed. A549 cells were collected stably transfected with sh-circ-MBOAT2 or sh-NC, and the cell suspension (5 × 10^7^ cells/mL) was inoculated into the subcutis of the axillae of the mice at 0.1 mL per mouse. A 7-day measurement was taken of the length and width of the tumors: (length × width^2^)/2. The growth curve of the subcutaneous grafted tumor in nude mice was plotted. All nude mice were put to death at 28 d, and the tumors were weighed with an electronic balance and photographed.

The tumor samples were preserved in a 4% paraformaldehyde solution, methodically dehydrated, encased in paraffin, and sliced into sections of 4 μm thickness. Sections were routinely dewaxed and hydrated, endogenous peroxidase was eliminated by H_2_O_2_, antigen was repaired by microwave, 10% normal goat serum was blocked for 30 min, and Ki-67 (1:100, Abcam), E-cadherin (1:200, Abgent, USA), and N-cadherin (1:200, Affinity, USA) antibodies were dropped for incubation. The stained sections were stained with brownish-yellow granules in the field of view of microscope magnification of 400 ×. Ten fields of view were randomly selected for taking photographs and images were analyzed using the Image J software.

### Statistical analysis

Every piece of data was analyzed by GraphPad Prism 9.0 (GraphPad Software, USA), with the results presented as mean ± standard deviation. Correlation analysis employed Pearson analysis, while the link between circ-MBOAT2 and NSCLC patients’ clinicopathological traits was ascertained using the chi-square test. For contrasting two groups, the t-test was employed, while for multi-group comparisons, one-way ANOVA and Tukey’s multiple comparison test were utilized. The statistical significance of the differences was evident as *P* < 0.05.

## Results

### Circ-MBOAT2 expression is upregulated in NSCLC

**Fig. 1:**
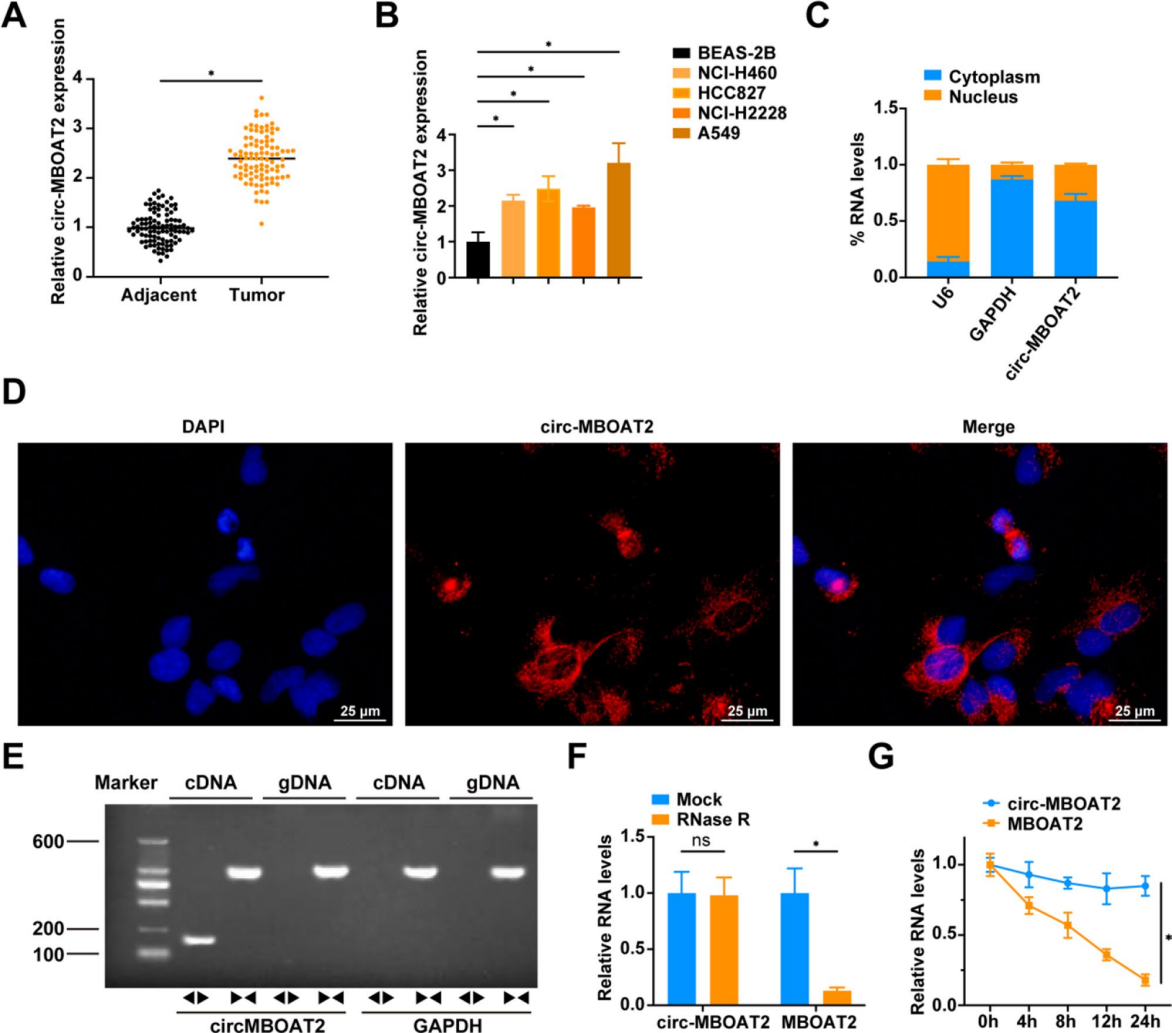
Circ-MBOAT2 expression is upregulated in NSCLC **A**: RT-qPCR to detect circ-MBOAT2 in NSCLC tissues and paracancerous tissues (*N* = 97); **B**: RT-qPCR to detect circ-MBOAT2 in BEAS-2B and the human NSCLC cell lines; **C**: RNA levels of circ-MBOAT2, GAPDH and U6 in the nucleus and cytoplasm of A549 cells; **D**: RNA FISH analysis of circ-MBOAT2 in A549 cells; **E**: PCR analysis of circMBOAT2 by divergent primers and convergent primers in cDNA and gDNA; **F**: RNase R assay to validate the circular structure of circMBOAT2; **G**: Actinomycin D assay to validate the circular structure. * *P* < 0.05. ns, not significant. Each experiment in this work was conducted independently at least three times

We collected 97 NSCLC tissues and corresponding paracancerous tissues for testing and found that circ-MBOAT2 expression was up-regulated in NSCLC tissues (Fig. 1A). A chart presenting the clinicopathological characteristics of 97 NSCLC patients can be found in Table 1. NSCLC patients were grouped based on median circ-MBOAT2 expression into two groups: low expression and high expression. Circum-MBOAT2 up-regulation correlated with tumor size, differentiation, TNM stage, and lymph node metastasis in NSCLC (Table 1). RT-qPCR assay showed (Fig. 1B) that circ-MBOAT2 expression was greater in NSCLC cell lines than in BEAS-2B cells, with A549 cells showing the most pronounced increase, making them the focus of further experiments. As a result of nucleoplasmic separation and FISH assay, circMBOAT2 was localized predominantly in the nucleus of A549 (Fig. 1C, D). According to PCR analysis, circ-MBOAT2 can only be amplified in cDNA by divergent primers, whereas it is not amplified in gDNA (Fig. 1E). Resistance to RNase R indicated a closed-loop structure of circ-MBOAT2 (Fig. 1F). Actinomycin D treatment revealed that circMBOAT2 remained stable compared to MBOAT2 mRNA (Fig. 1G).

### Silencing circ-MBOAT2 inhibits NSCLC cell proliferation, EMT, and immune escape

**Fig. 2:**
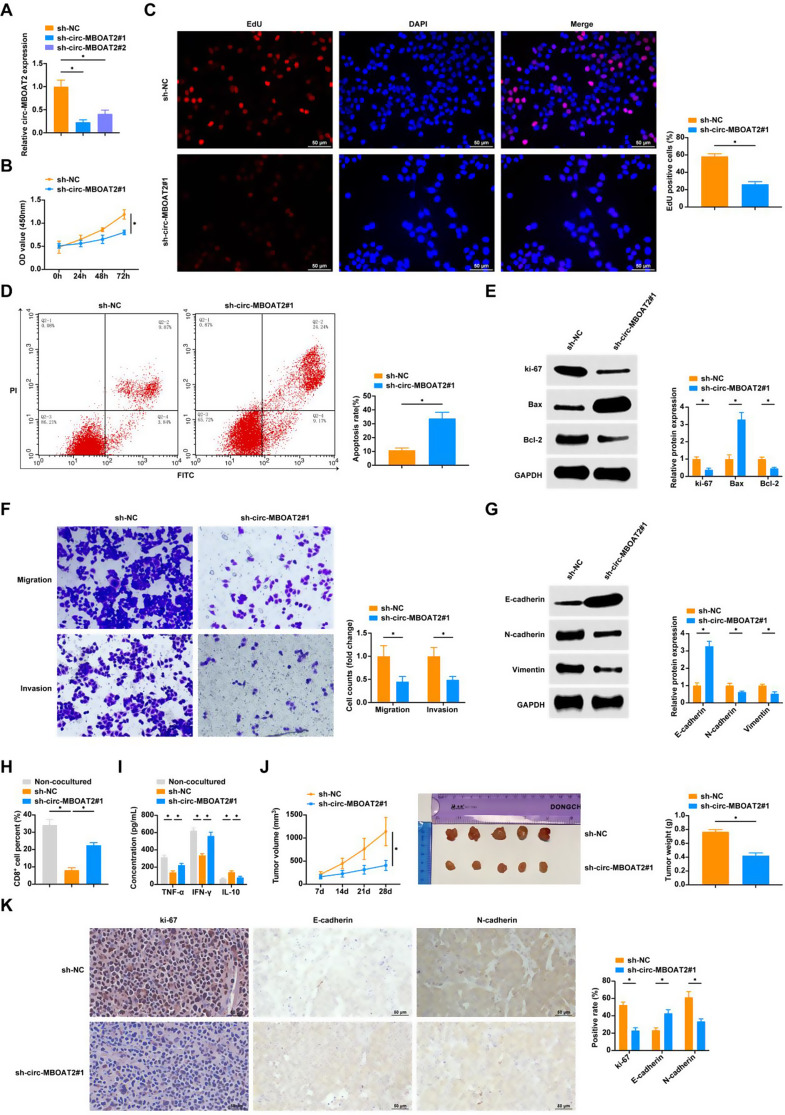
Knockdown of Circ-MBOAT2 inhibits NSCLC cell proliferation, EMT and immune escape **A**: RT-qPCR to detect circ-MBOAT2; **B**-**C**: CCK8 assay and EdU assay to detect cell proliferation; **D**: AnnexinV-PI double staining to detect apoptosis rate; **E**: Western blot to detect the protein expression level of cellular Ki-67, Bax, and Bcl-2; **F**: Transwell assay to detect cell migration and invasion; **G**: Western blot to detect protein expression levels of cellular E-cadherin, N-cadherin and Vimentin; **H**: Flow cytometry detection of the percentage of CD8^+^ T cells in PBMCs; **I**: ELISA detection of TNF-α, IFN-γ, and IL-10 in the supernatant of PBMCs; **J**: Comparison of transplanted tumors volumes and weights in nude mice (*N* = 5); **K**: Immunohistochemical assessment of Ki67, E-cadherin and N-cadherin in nude mice transplanted tumor tissues (*N* = 5). * *P* < 0.05. ns, not significant. Each experiment in this work was conducted independently at least three times

circ-MBOAT2 was knocked down in A549 cells by transfection of sh-circ-MBOAT2#1 and sh-circ-MBOAT2#2. The higher knockdown efficiency of sh-circ-MBOAT2#1 led to its selection for subsequent experiments (Fig. 2A). By CCK-8 and EDU experiments, we found that silencing circ-MBOAT2 hampered NSCLC cell proliferation (Fig. 2B, C), which was further verified by the elevated expression of the proliferative protein Ki-67 (Fig. 2E). Transfection of sh-circ-MBOAT2#1 significantly increased the apoptosis rate and Bax level and reduced Bcl-2 level in tumor cells (Fig. 2D, E). Transwell assay results, on the other hand, showed that silencing circ-MBOAT2 hampered the migration and invasion of tumor cells (Fig. 2F). In Western blot analysis, circ-MBOAT2 silencing reduced N-cadherin and Vimentin protein levels while increasing E-cadherin protein levels (Fig. 2G).

A549 cells co-cultured with PBMCs decreased their CD8^+^ T cell proportion, TNF-α and IFN-γ production in the supernatants, and elevated IL-10 production. Co-culture with A549 cells transfected with sh-circ-MBOAT2#1, however, led to an increase in CD8^+^ T cells and TNF-α and IFN-γ levels, as well as a decrease in IL-10 levels in PBMCs. This indicated that inhibition of circ-MBOAT2 blocked the immune escape of NSCLC cells (Fig. 2H, I).

For assessing circMBOAT2’s biological role in a living organism, a mouse xenograft tumor model was created. Findings verified a notable reduction in tumor growth following circMBOAT2 downregulation (Fig. 2J). Immunohistochemical staining of the tumor tissues taken afterwards revealed that Ki67 and N-cadherin proteins in the group with down-regulated circMBOAT2 expression was weakened, while E-cadherin protein was enhanced (Fig. 2K).

### Circ-MBOAT2 competitively binds to miR-664b-3p

**Fig. 3:**
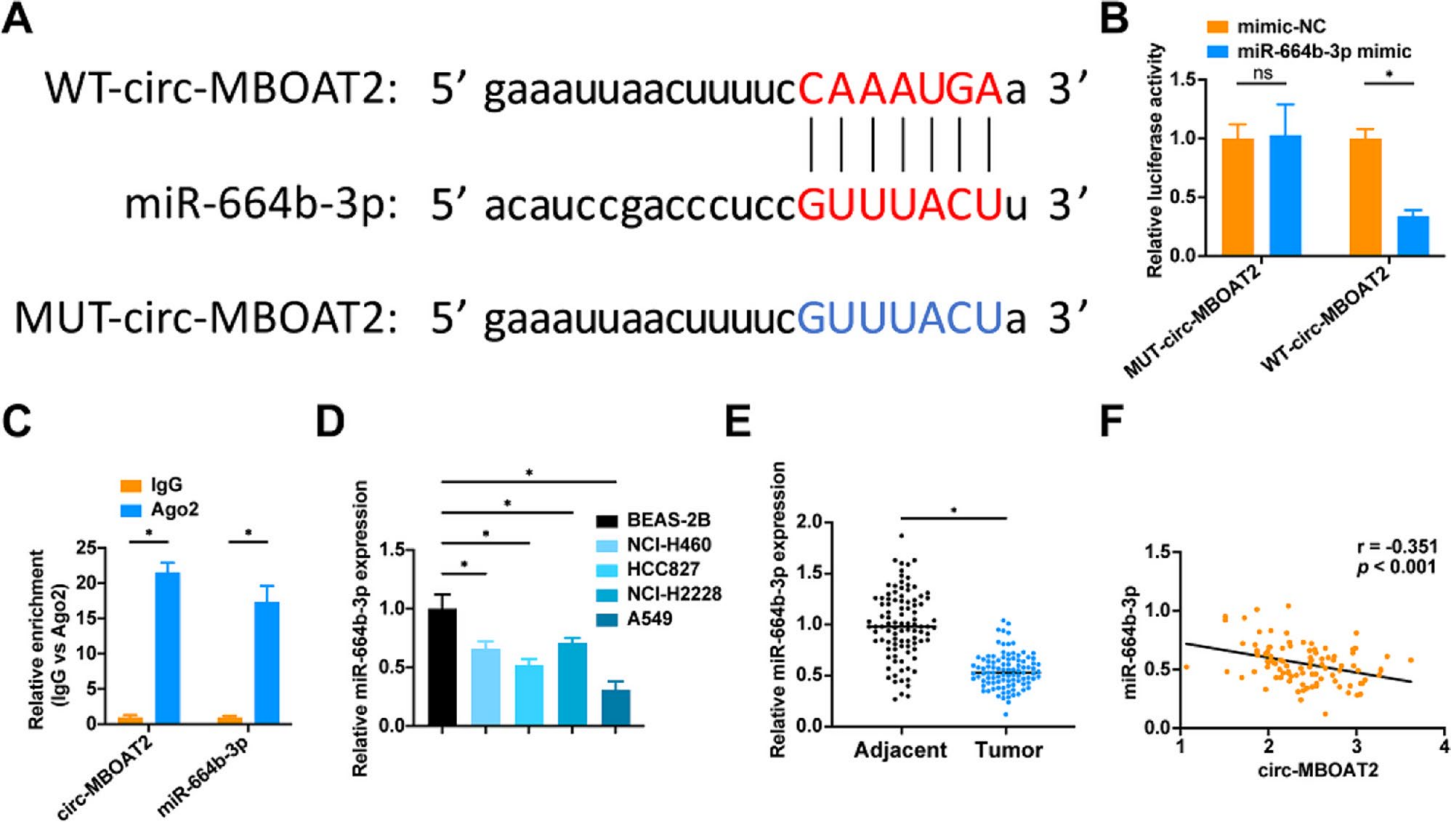
Circ-MBOAT2 competitively binds to miR-664b-3p **A**: Bioinformatics website ENCORI (https://rnasysu.com/encori/index.php) predicted the binding sites of circ-MBOAT2 and miR-664b-3p; **B**: Luciferase reporter gene assay to verify the binding relationship between circ-MBOAT2 and miR-664b-3p; **C**: RIP to verify the binding relationship between circ-MBOAT2 and miR-664b-3p; **D**: RT-qPCR to detect miR-664b-3p in BEAS-2B and the human NSCLC cell lines; E: RT-qPCR to detect miR-664b-3p in NSCLC tissues and paracancerous tissues (*N* = 97); F: Pearson’s test to analyze the correlation between NSCLC patients’ circ-MBOAT2 and the miR-664b-3p expression level (*N* = 97). * *P* < 0.05. ns, not significant. Each experiment in this work was conducted independently at least three time

We investigated circ-MBOAT2’s role as a miRNA sponge in NSCLC by taking advantage of its cytoplasmic localization. Based on online analysis software, a specific binding region between circ-MBOAT2 and miR-664b-3p was predicted (Fig. 3A). In A549 cells, circ-MBOAT2-WT and miR-664b-3p mimic cotransfected cells showed decreased luciferase activity (Fig. 3B). RIP experiments further validate the binding relationship between circ-MBOAT2 and miR-664b-3p (Fig. 3C). miR-664b-3p showed down-regulation of its expression in both NSCLC tissues and cells (Fig. 3D, E), and analyses revealed that miR-664b-3p and circ-MBOAT2 levels in NSCLC tissues were negatively correlated (Fig. 3F).

### Circ-MBOAT2 knockdown inhibits NSCLC cell proliferation, EMT, and immune escape by promoting miR-664b-3p expression

**Fig. 4:**
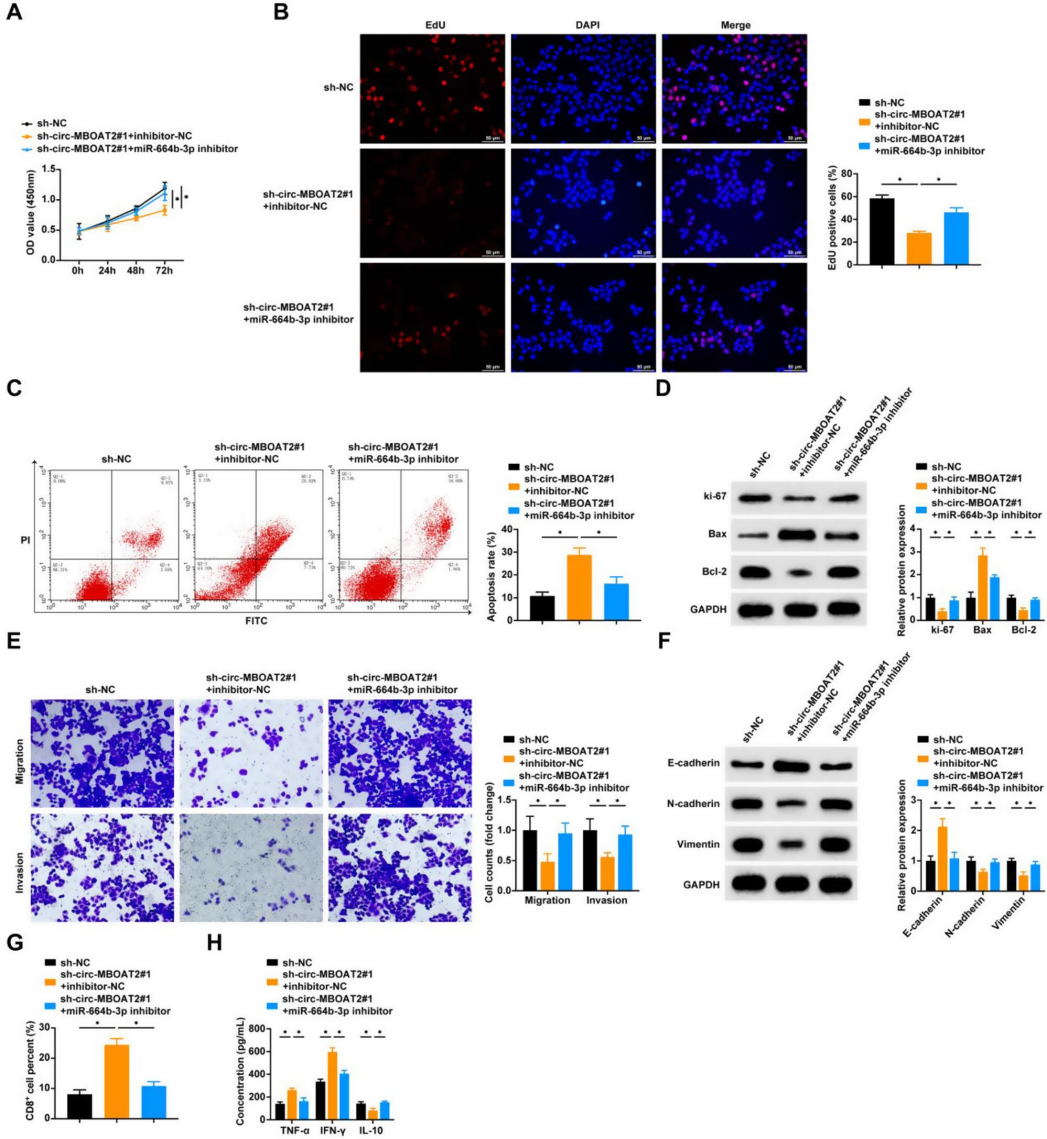
Knockdown of circ-MBOAT2 inhibits NSCLC cell proliferation, EMT and immune escape by promoting miR-664b-3p expression **A**-**B**: CCK8 assay and EDU assay to detect cell proliferation; **C**: AnnexinV-PI double staining assay to detect apoptosis rate; **D**: Western blot assay to detect protein expression levels of cellular Ki-67, Bax and Bcl-2; **E**: Transwell assay to detect cell migration and invasion; **F**: Western blot assay to detect cellular E-cadherin, N-cadherin and Vimentin protein expression levels; **G**: Flow cytometry to detect the percentage of CD8^+^ T cells in PBMCs; **H**: ELISA to detect the levels of TNF-α, IFN-γ, and IL-10 in the supernatant of PBMCs. * *P* < 0.05. ns, not significant. Each experiment in this work was conducted independently at least three time

We performed cell function experiments after co-transfecting sh-circ-MBOAT2#1 and miR-664b-3p inhibitor into A549 cells, and the experimental results showed that inhibition of miR-664b-3p impaired the suppression of NSCLC cell proliferation and Ki-67 expression levels by knockdown of circ-MBOAT2 (Fig. 4A, B, D). Comparing cells transfected with inhibitor-NC and sh-circ-MBOAT2, apoptosis rate and Bax protein expression were reduced and anti-apoptotic protein Bcl-2 expression was elevated in cells transfected with sh-circ-MBOAT2#1 and miR-664b-3p inhibitor (Fig. 4C, D). In addition, miR-664b-3p inhibitor promoted the migration and invasion of tumor cells (Fig. 4E), as well as N-cadherin and Vimentin, and reduced E-cadherin (Fig. 4F). Co-culture of PBMCs with A549 cells revealed that compared to the sh-circ-MBOAT2#1 + inhibitor-NC group, sh-circ-MBOAT2#1 + miR-664b-3p inhibitor group showed a decrease in the percentage of CD8^+^ T cells, TNF-α and IFN-γ levels, and an up-regulation of IL-10 (Fig. 4G, H).

### TLK1 is mediated by miR-664b-3p

**Fig. 5:**
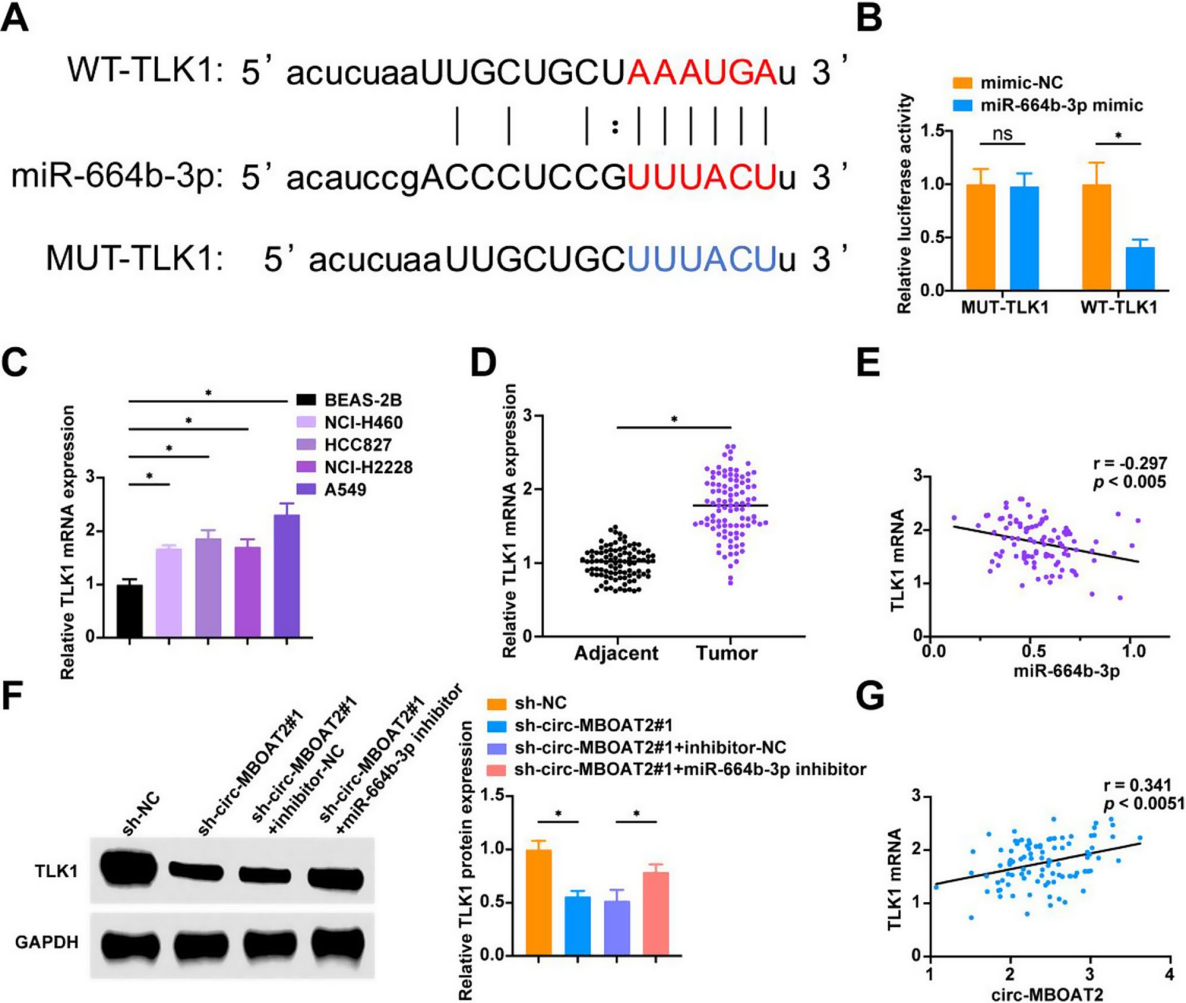
TLK1 is a target gene of miR-664b-3p **A**: Bioinformatics website ENCORI (https://rnasysu.com/encori/index.php) predicted the binding site of miR-664b-3p and TLK1; **B**: Luciferase reporter gene assay to verify the binding relationship between miR-664b-3p and TLK1; **C**: RT-qPCR and Western blot assay to detect TLK1 in BEAS-2B and human NSCLC cell lines; **D**: RT-qPCR to detect TLK1 mRNA expression level in NSCLC tissues and paracancer tissues (*N* = 97); **E**: Pearson test to analyze the correlation between TLK1 mRNA expression level and miR-664b-3p expression level in NSCLC patients (*N* = 97); **F**: Western blot to detect TLK1 protein expression level in A549 cells after transfection; **G**: Pearson test to analyze the correlation between TLK1 mRNA expression level and circ-MBOAT2 expression level (*N* = 97). * *P* < 0.05. ns, not significant. Each experiment in this work was conducted independently at least three time

Bioinformatics software predicted a targeting relationship between miR-664b-3p and TLK1 (Fig. 5A). In luciferase activity assays, TLK1-WT cells co-transfected with miR-664b-3p mimic showed significantly reduced relative activity, but TLK1-MUT cells did not (Fig. 5B). TLK1 showed up-regulated expression in both NSCLC tissues and cells (Fig. 5C, D), and a negative correlation was observed between miR-664b-3p and TLK1 expression levels in NSCLC tissues (Fig. 5E). Downregulating circ-MBOAT2 inhibited TLK1 expression, and this inhibition was mitigated by miR-664b-3p inhibitor (Fig. 5F), and circ-MBOAT2 and TLK1 levels in NSCLC tissues were positively correlated (Fig. 5G).

### Silencing TLK1 inhibits NSCLC cell proliferation, EMT, and immune escape

**Fig. 6:**
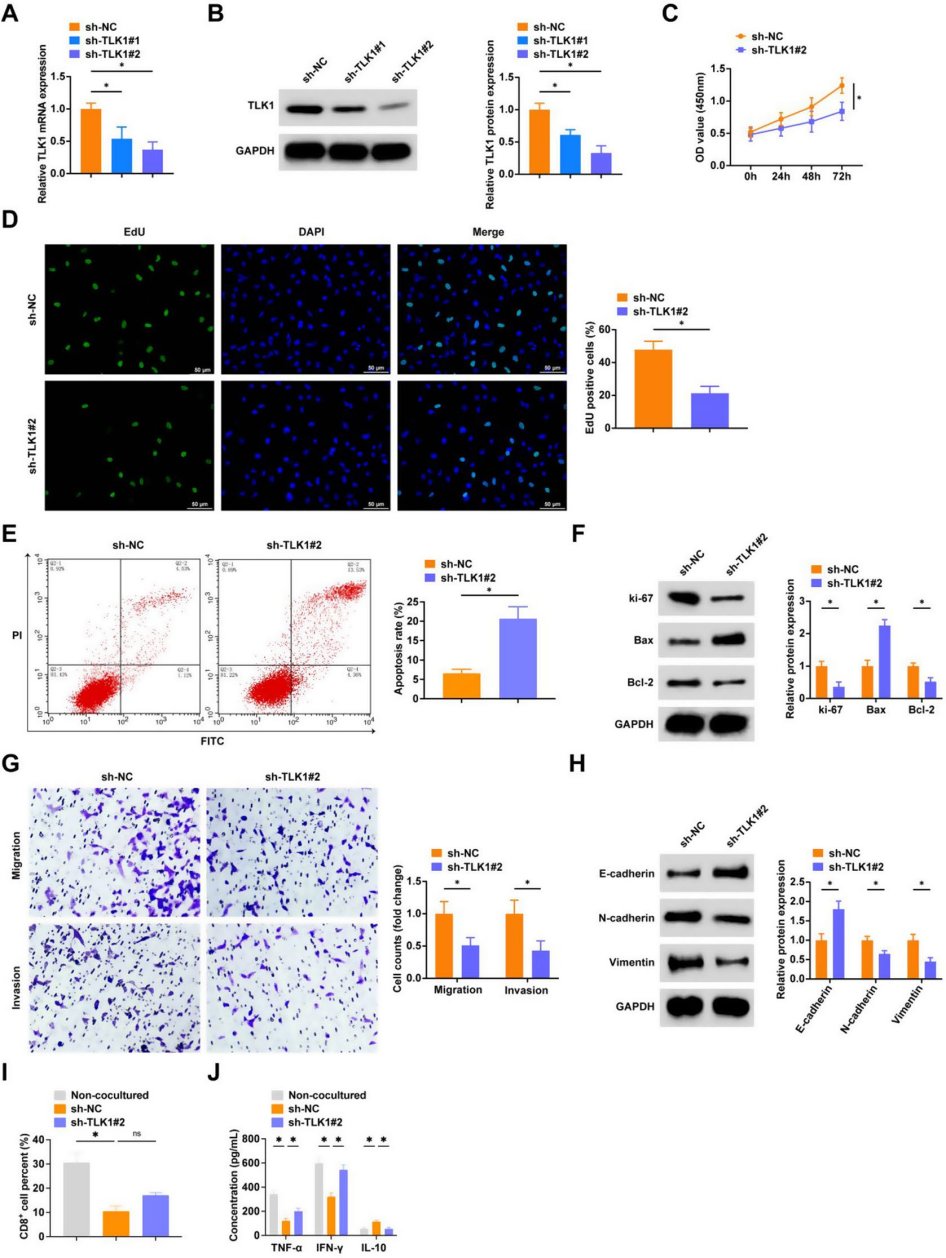
Knockdown of TLK1 inhibits NSCLC cell proliferation, EMT and immune escape **A**: RT-qPCR to detect cellular TLK1 mRNA expression level; **B**: Western blot to detect cellular TLK1 protein expression level; **C**-**D**: CCK8 assay and EDU assay to detect cell proliferation; **E**: AnnexinV-PI double staining assay to detect apoptosis rate; **F**: Western blot to detect the protein expression level of cellular Ki-67, Bax, and Bcl-2; **G**: Transwell assay to detect cell migration and invasion; **H**: Western blot to detect protein expression levels of cellular E-cadherin, N-cadherin and Vimentin; I: Flow cytometry to detect the percentage of CD8^+^ T cells in PBMCs; **J**: ELISA to detect TNF-α, IFN-γ, and IL-10 in the supernatant of PBMCs.* *P* < 0.05. ns, not significant. Each experiment in this work was conducted independently at least three time

TLK1 was knocked down in A549 cells through transfection with sh-TLK1#1 and sh-TLK1#2. The higher knockdown efficiency of sh-TLK1#1 led to its selection for subsequent experiments (Fig. 6A, B). CCK-8 and EDU assays revealed that TLK1 downregulation significantly blocked NSCLC cell proliferation (Fig. 6C, D), a finding corroborated by reduced expression of the proliferation marker Ki-67 (Fig. 6F). Transfection with sh-TLK1#1 markedly increased the apoptosis rate and Bax protein levels while decreasing Bcl-2 expression in tumor cells (Fig. 6E, F). Transwell assays demonstrated that TLK1 silencing impaired tumor cell migration and invasion (Fig. 6G). Further analysis by Western blot demonstrated that TLK1 knockdown lowered N-cadherin and Vimentin levels and raised E-cadherin expression (Fig. 6H).

In co-culture experiments with sh-TLK1#1-transfected cells and PBMCs, we observed an increased percentage of CD8^+^ T cells, elevated TNF-α and IFN-γ levels in PBMC supernatants, and a significant reduction in IL-10 (Fig. 6I, J).

### miR-664b-3p blocks NSCLC cell proliferation, EMT and immune escape by targeting TLK1

**Fig. 7:**
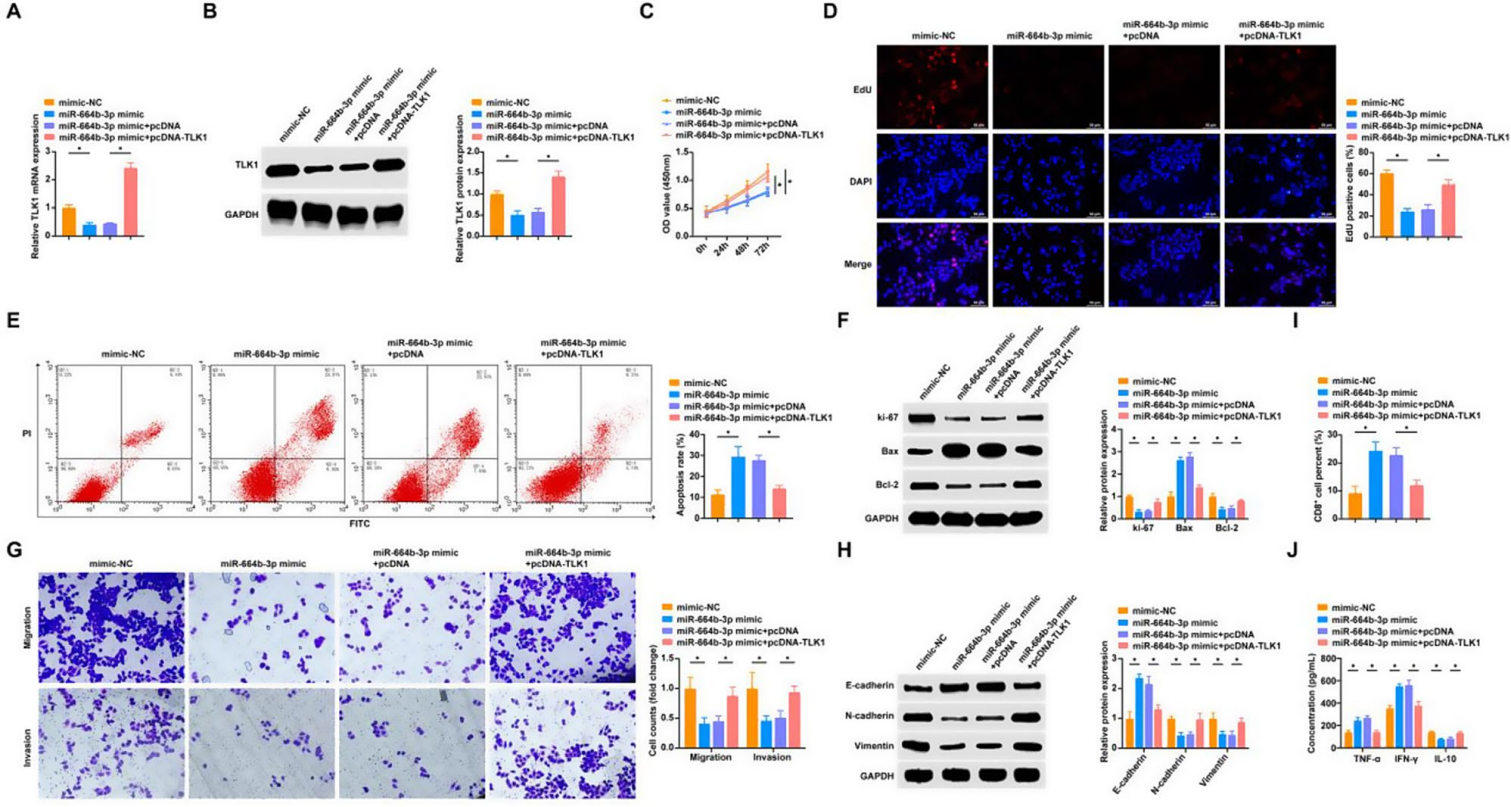
miR-664b-3p inhibits NSCLC cell proliferation, EMT and immune escape by targeting TLK1 **A**: RT-qPCR to detect cellular TLK1 mRNA expression level; **B**: Western blot to detect cellular TLK1 protein expression level; **C**-**D**: CCK8 assay and EDU assay to detect cell proliferation; **E**: AnnexinV-PI double staining assay to detect apoptosis rate; **F**: Western blot to detect cellular Ki-67, Bax and Bcl-2; **G**: Transwell assay to detect cell migration and invasion; **H**: Western blot to detect cellular E-cadherin, N-cadherin and Vimentin; **I**: Flow cytometry to detect the percentage of CD8^+^ T cells in PBMCs; **J**: ELISA to detect TNF-α, IFN-γ, and IL-10. * *P* < 0.05. ns, not significant. Each experiment in this work was conducted independently at least three time

RT-qPCR verified the transfection efficiency of miR-664b-3p mimic, and Western blot confirmed that miR-664b-3p mimic inhibited TLK1 protein, while pcDNA-TLK1 successfully this inhibition effect (Fig. 7A, B). Overexpressing miR-664b-3p was found to inhibit cell proliferation and promoted apoptosis (Fig. 7C-F). pcDNA-TLK1 could reverse these effects. Additionally, miR-664b-3p upregulation hampered migration and invasion of tumor cells (Fig. 7G, H). In contrast, these effects of upregulating miR-664b-3p on tumor cells were mitigated by overexpressing TLK1. Similarly, co-culture of cells transfected with miR-664b-3p mimic and PBMCs resulted in a decrease in the percentage of CD8^+^ T cells, a significant decrease in TNF-α and IFN-γ in the supernatants of PBMCs, and a significant increase in IL-10 (Fig. 7I, J). In contrast, pcDNA-TLK1 weakened the role of miR-664b-3p mimic when co-cultured with PBMCs.

## Discussion

NSCLC is a highly heterogeneous tumor type with three main histological subtypes. A steady increase in survival rates has been observed over the past few decades thanks to novel therapies such as targeted therapies and immunotherapies, but long-term survival rates remain low.

Immunotherapy focused on the host immune response shows significant potential for treating NSCLC [28]. However, the tumor microenvironment of NSCLC allows tumors to evade immune attack during immunotherapy [7]. CircRNAs serve as predictive biomarkers and immunotherapeutic targets for immunotherapy [29, 30]. Studies related to the involvement of circRNAs in immune escape of NSCLC cells are also common [31]. Previous research has established that circ-MBOAT2 demonstrates dysregulated expression in multiple malignancies, functioning as an oncogenic driver that facilitates tumorigenesis and accelerates malignant progression. However, the role of circ-MBOAT2 in tumor immune escape is still obscure. Our study matched those of prior studies and also indicated that circ-MBOAT2 expression was upregulated in NSCLC patients, and that knocking down circ-MBOAT2 hampered proliferation of NSCLC cells, as well as EMT and blocked the immune escape of NSCLC cells. Mechanistically, circ-MBOAT2 bound to miR-664b-3p to enhance TLK1 expression and subsequently induced NSCLC cell proliferation, EMT and immune escape.

Recent studies identifies miR-664b-3p as a tumor-suppressive miRNA in cancer pathogenesis, including NSCLC [32, 33]. This study demonstrated that circ-MBOAT2 directly sponges miR-664b-3p and represses its expression, with rescue experiments confirming that circ-MBOAT2’s biological functions are mechanistically dependent on miR-664b-3p. Our findings establish that circ-MBOAT2 drives miR-664b-3p dysregulation and functions as a critical upstream regulator of miR-664b-3p in NSCLC pathogenesis.

Tousled kinase (TSL) is essential for plant leaf and flower development, and a homologue of TSL was later identified and named TLK [34]. The human TLK family contains two genes, TLK1 located at 2q31.1 and TLK2 at 17q23.2, and the coding sequences of the two share 84% identity at the amino acid level, with kinase structural domains that can be up to 96% similar [35]. TLK is a highly conserved serine/threonine protein kinase involved in cytological processes such as DNA replication, transcription, damage repair, chromatin assembly, and chromosome segregation [36]. TLK1 is known to be highly expressed in several tumors and acts as an oncogene, aiding in cancer development and progression [37, 38]. Particularly, a previous investigation identified tertiary amine-linked indirubin-3’-oximes as potent anticancer agents targeting TLK activity, which exhibited marked growth-inhibitory effects on NSCLC A549 cells through TLK1 pathway suppression [39]. In the present study, we demonstrated that TLK1 expression was up-regulated in NSCLC samples. TLK1 was validated as a target gene of miR-664b-3p and could be positively regulated by circ-MBOAT2. TLK1 knockdown weakened NSCLC cell proliferation, EMT, and immune escape capabilities. Conversely, TLK1 overexpression rescued the inhibitory effects of miR-664b-3p on NSCLC cell malignant behaviors.

TLKs safeguard telomeric integrity and stabilize repetitive genomic elements, with their genetic ablation inducing telomere-selective elongation while potently activating innate immune surveillance pathways. Elevated TLK expression in malignancies correlates with chromosomal instability and immune evasion mechanisms. Mechanistically, TLKs preserve the structure of chromatin at recurring genomic sites and telomeres. The reduction of TLK leads to the destabilization of heterochromatin and encourages the alternative lengthening of telomeres (ALT), thereby initiating the cGAS-STING-TBK1-driven pathways of innate immune signaling. These insights establish TLKs as pharmacologically exploitable targets in ALT-driven tumors and chromosomally unstable cancers, while providing mechanistic context for understanding how the circ-MBOAT2 coordinates immune evasion in NSCLC pathogenesis through TLK1-dependent regulation [40].

Despite our findings, this study has some limitations. Firstly, while the A549 cell line serves as a well-characterized experimental model for mechanistic interrogation, expanding investigations to other molecularly distinct NSCLC cell lines (e.g., squamous cell carcinoma H1703, EGFR-mutant PC-9, and KRAS-driven H358) is imperative to assess the pan-subtype relevance of the circ-MBOAT2/miR-664b-3p/TLK1 regulatory axis in NSCLC pathogenesis. Secondly, the molecular determinants orchestrating circ-MBOAT2 biogenesis and the downstream signaling cascades executing TLK1-mediated oncogenic functions remain to be systematically delineated, highlighting the imperative for future mechanistic investigations. Lastly, multi-center validation studies incorporating comprehensive histopathological classifications and molecular profiling are needed to conclusively establish the prognostic biomarker potential of circ-MBOAT2.

## Conclusion

Circ-MBOAT2 expression is upregulated in NSCLC, promoting TLK1 expression via miR-664b-3p, which accelerates NSCLC proliferation, EMT, and immune escape. Circ-MBOAT2 may serve as a potential clinical target for treating NSCLC, based on these data.

## Data Availability

The datasets used and/or analyzed during the present study are available from the corresponding author on reasonable request.
